# Hypersensitivity Vasculitis with Leukocytoclastic Vasculitis Associated with Alpha-1-Proteinase Inhibitor

**DOI:** 10.1155/2009/941258

**Published:** 2010-02-24

**Authors:** Nicola W. Mwirigi, Charles F. Thomas

**Affiliations:** Thoracic Diseases Research Unit, Division of Pulmonary, Critical Care and Internal Medicine, Department of Medicine, Mayo Clinic College of Medicine, Rochester, MN 55905, USA

## Abstract

Prolastin is a commercially available form of alpha-1-antitrypsin (AAT) that is derived from pooled human plasma and used for treatment of severe alpha-1-antitrypsin deficiency (AATD). We describe a patient with AATD who developed presumed hypersensitivity vasculitis (HV) following a Prolastin infusion. Hypersensitivity vasculitis (HV), or cutaneous vasculitis, is characterized by inflammation of the small vessels of the skin with resultant ischemia to the distally supplied areas. To our knowledge, this is the first reported case of presumed hypersensitivity vasculitis following Prolastin infusion.

## 1. Case Report

A-56-year old woman with a background history of type 2 diabetes, hypertension, GERD, severe asthma, COPD, and newly diagnosed alpha-1-antitrypsin deficiency (AATD) (ZZ phenotype) acutely developed shortness of breath and fatigue following her first Prolastin infusion. A week later she received a second infusion with premedication. Over the following 3 weeks the patient complained of a constellation of symptoms including dyspnea, wheezing and one episode of hemoptysis. She also complained of nonspecific arthralgias and a diffuse rash. During this time she was admitted to her home hospital where she was treated with steroids and antibiotics (Augmentin). She did not improve and was subsequently transferred to Mayo Clinic for further evaluation. 

**Figure 1:**
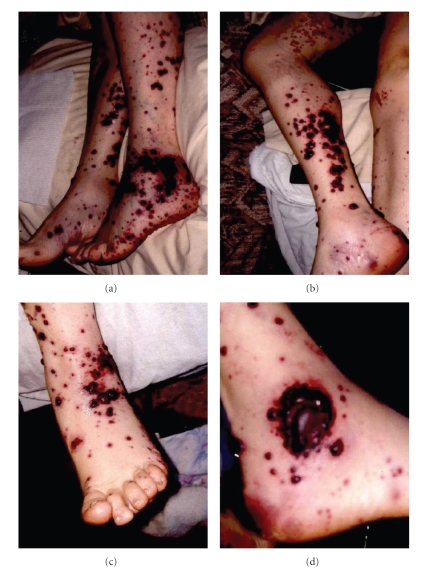
Photographs of the vasculitic lesions on the lateral (a), posterior (b), and anterior aspects of the legs. Close-up of the large ulceration on the lateral malleolus (d).

The initial evaluation revealed the following: temperature, 37.0°C; blood pressure, 132/78; pulse rate, 73; oxygen saturations, 98% on 3 L/min nasal cannula; and respiratory rate, 18. She was alert and oriented and sitting comfortably in no apparent distress with a cushingoid appearance (due to steroid use for chronic asthma and COPD). Physical exam revealed multiple palpable purpuric violaceous papules coalescing into serpiginous plaques with large adherent black eschars overlying her upper and lower extremities. Several punched out ulcerations with yellow fibrinous adherent material were present over her bilateral upper thighs and buttocks (Figure 1). Lung exam revealed bibasilar crackles. Remainder of her exam was normal. A complete list of her medications is included in Table 2. 

Laboratory studies showed a normocytic anemia, slight leucocytosis, elevated sedimentation rate, C-reactive protein, and low AAT (Table 1). Urinalysis was unremarkable. CT chest showed consolidative infiltrates in the left lower lung posteriorly and scattered alveolar infiltrates throughout both lungs. Bronchoscopy revealed old blood in the R lower lobe subsegmental bronchus without any other endobronchial lesions. BAL had a bloody return which cleared suggestive of prior bleeding. Skin punch biopsy of the left lower leg revealed mild superficial dermal perivascular neutrophilic inflammation. Direct immunofluorescence of the skin biopsy was negative for deposits (IgG, IgM, IgA, C3, and fibrinogen all negative). She was treated with prednisone 60 mg orally once a day, dapsone 100 mg by mouth orally once a day for *Pneumocystis jiroveci* pneumonia prophylaxis as she had a sulfonamide allergy, daily whirl pool therapy, and topical therapy with Sweitzer solution (zinc-boron-salicylate solution) and 0.1% triamcinolone cream. Her skin lesions and associated symptoms continued to improve with treatment.

## 2. Discussion

Alpha-1-antitrypsin (AAT) is a 394 amino acid glycoprotein produced by the liver and normally found in the serum and extravascular locations, including the alveolar lining of the lung. The main function of AAT is inhibition of neutrophil elastase. If the quantity or functional activity of AAT is significantly reduced, the resulting imbalance leaves the alveolar walls susceptible to proteolytic damage, resulting in emphysema [1]. The association between AATD and heritable emphysema was discovered in 1963 [2, 3]. Subsequent epidemiologic evidence suggested that a serum AAT level of 11 *μ*mol/L (80 mg/dL), which is 35% of the normal level, represents a “threshold” value below which the risk of developing emphysema is significantly increased [1]. The ability to isolate and purify the AAT protein from human blood has made augmentation therapy possible. Augmentation therapy has been shown to raise antiprotease serum and epithelial lining fluid levels above the “protective threshold” value. Evidence suggests that this approach slows the decline in lung function, could reduce infection rates, might enhance survival, and is well tolerated [4]. Based on available evidence, the American Thoracic Society recommends augmentation therapy for individuals with both a documented severe deficiency of AAT and fixed airflow obstruction [1]. Prolastin, a commercial preparation of AAT from pooled blood, is given by intravenous infusion at a dose of 60 mg/kg ideal body weight once a week. The largest cohort study in the USA looking at side effects was conducted by the National Heart Lung and Blood institute (NHLBI) AATD Registry Group. The most frequent reported side effects include headache, dizziness, nausea, and dyspnea [5]. Although acute allergic reactions, including anaphylaxis, have been reported, these events are rare. It is important to be aware that patients with severe AAT deficiency may also be IgA deficient and that Prolastin contains some IgA [5]. 

Possible differentials for this patient's skin findings are discussed below. Necrotizing panniculitis, which is characterized by inflammatory lesions of the skin and subcutaneous tissue, is the major dermatologic manifestation of AATD [6–9]. AATD individuals generally present with a hot, painful, red subcutaneous nodule or plaque on the thigh or buttocks [10]. These lesions can be difficult to distinguish from panniculitis due to other causes, but may be more inflammatory with an oily yellow discharge and more pronounced histologic evidence of acute inflammation. Panniculitis is the least common of the well-recognized complications of AAT deficiency, with fewer than 50 cases reported in the English literature [6, 9–13]. The prevalence among AAT-deficient subjects is probably less than one case per thousand. Panniculitis has been reported to occur in a variety of phenotypes, including PI*ZZ, PI*MZ, PI*SS, and PI*MS. Seventy percent of reported cases have occurred in patients with PI*ZZ phenotype and severe AAT deficiency [10]. Similar to the pathophysiology of emphysema in such individuals, the panniculitis is thought to result from unopposed proteolysis in the skin. Deep excisional biopsies may be required to establish the diagnosis. Characteristic histologic findings include lobular fat necrosis of the lower reticular dermis, with abundant neutrophil influx interspersed with normal-appearing fat and necrotic panniculus [14]. Based upon this presumed pathophysiologic mechanism of unopposed proteolysis, treatment of panniculitis in AATD subjects has focused on restoring antiprotease activity. Intravenous infusion of purified AAT has ameliorated the panniculitis in some patients [7, 10]. Other treatments have included dapsone (100 mg/day for several weeks) and doxycycline (200 mg/day for as long as several months) [7, 10, 12]. The latter is believed to act by scavenging reactive oxygen species produced by neutrophils and/or by slowing the breakdown of matrix proteins by elastase. Of note the appearance of these skin findings in this case is not classic for panniculitis. 

Wegener's Granulomatosis, which is a systemic vasculitis of the medium and small arteries and the venules and arterioles, can have dermatological manifestations. These include vesicular, palpable purpuric, ulcerative, and hemorrhagic lesions. This patient's presentation and clinical findings are not compatible with the American College of Rheumatology criteria for the classification of Wegener's granulomatosis [15]. In particular, there was no upper airway involvement, no evidence of granulomatous inflammation on her biopsy, no abnormal urinary sediment or renal involvement, and negative PR3 and MPO antibodies.

Likewise this patient's presentation and findings are not compatible with Churg-Strauss syndrome (CSS), also called allergic granulomatosis and angiitis. This multisystem disease is characterized by allergic rhinitis, asthma, and prominent peripheral blood eosinophilia, none of which were present in this case. Two-thirds of patients with CSS have skin lesions, which usually appear as subcutaneous nodules on the extensor surfaces of the arm, particularly the elbows, hands, and legs [16]. The skin lesions can appear as palpable purpura, macular or papular erythematous rash, hemorrhagic lesions, ranging from petechiae to extensive ecchymoses, tender cutaneous or subcutaneous nodules in which granulomas are usually found on biopsy.

Hypersensitivity vasculitis (HV) or cutaneous vasculitis is characterized by inflammation of the small vessels of the skin with resultant ischemia to the distally supplied areas [6]. In 1990 the American College of Rheumatology proposed the following five criteria for the classification of hypersensitivity vasculitis:

1. age >16,
2. use of possible offending drug in a temporal relation to the symptoms,
3. palpable purpura,
4. maculopapular rash,
5. biopsy of skin lesion showing neutrophils around an arteriole or venule.

The presence of three or more of these criteria had a sensitivity and specificity for the diagnosis of hypersensitivity vasculitis of 71 and 84 percent, respectively [4]. Leukocytoclastic vasculitis (LV) is a histologic diagnosis. It involves the deposition of immune complexes in vessel walls, ultimately leading to cellular infiltrates, cytokine release, and vessel damage. If IgA deposition is noted on skin biopsy, it is necessary to rule out Henoch-Schönlein purpura. Immunofluorescence can be used if the skin biopsy is inconclusive. Common causes of HV include drugs, infections (HIV, hepatitis B and C), malignancy, connective tissue disease (SLE, RA), and autoimmune disease. The most common offending drugs include antibiotics (penicillins, cephalosporins, and macrolides), loop and thiazide diuretics, sulfonamides, allopurinol, and phenytoin [17]. The major clinical findings of HV in addition to the skin lesions, palpable purpura and/or petechia, include fever, urticaria, arthralgias, lymphadenopathy, low serum complement levels, and an elevated erythrocyte sedimentation rate. In most patients, these symptoms and/or findings begin from 7 to 10 days after antigen exposure, the time required to produce a sufficient quantity of antibody to produce antigen-antibody complexes [18, 19]. However, the latent period may be as short as two to seven days with a secondary antigen exposure or longer than two weeks with a long-acting drug such as benzathine penicillin [20]. The initial laboratory work-up for evaluation of small vessel vasculitis has been suggested to include complete blood count, erythrocyte sedimentation rate, urinalysis, chemistry panel, stool guaiac, ANA, ANCA, Rheumatoid factor (RF), cryoglobulins, C3, C4, serology for hepatitis B and C, chest X-ray, and skin biopsy [21]. Skin biopsy with specimens for routine light microscopy and direct immunofluorescence examination is the first step in determining whether authentic vasculitis is present and excluding pseudovasculitic disorders (e.g., warfarin necrosis, cholesterol emboli). The choice and timing of the biopsy will affect diagnostic yields. Early lesions, less than 48 to 72 hours old, are more likely to yield neutrophilic vasculitis, whereas older lesions are more likely to show a solely perivascular lymphocytic infiltrate without evidence of vessel damage. Additional tests to consider include blood cultures and echocardiography if the patient has a high fever and/or has a heart murmur and anti-streptolysin O titers in children [22]. The treatment options include discontinuation of the inciting drug or antigen which should lead to resolution of the signs and diagnosis within a period of days to a few weeks. Conservative measures include rest, leg elevation, support hoses, avoiding cold exposure and tight fitting clothes [23]. Treatment of the underlying infection, such as the administration of interferon and ribavirin to those with hepatitis C virus and cryoglobulinemia, is recommended. In patients with more severe or persistent cutaneous disease not due to infection, drugs such as colchicine, antihistamines, and dapsone may be helpful [24–26]. Occasionally, combinations of these drugs (e.g., dapsone and pentoxifylline) are more effective than single therapy [27]. Patients with complicated or systemic disease in whom more toxic therapies are required should be referred to a rheumatologist for consultation. Immunosuppressive therapy with corticosteroids or cytotoxic agents should be reserved for the infrequent patient with fulminant or progressive disease, some of whom may have microscopic polyarteritis [26]. Novel therapy includes TNF-alpha inhibitors, intravenous immunoglobulins, or plasmapheresis [23]. Rechallenge of the suspected drug is not recommended.

Here we describe a patient who developed HV 2 weeks following her 2nd Prolastin infusion with fulminant skin disease. As mentioned previously drugs are the most common offending agents, but drug-induced vasculitis is a diagnosis of exclusion. It is hypothesized that drugs act as haptens that cause an immune response. The most common medications include antibiotics (penicillins, cephalosporins, macrolides), antiretrovirals, granulocyte colony-stimulating factor, loop and thiazide diuretics, sulfonamides, allopurinol, and phenytoin [28]. Furosemide, a medication reported to cause HV in a limited number of case based reports, was a stable medication for this patient and therefore unlikely to be implicated in her condition [29, 30]. Another important factor to consider is that Prolastin contains trace amount of polyethylene glycol (PEG), and other medications mixed with PEG have been reported to cause HV (such as PEG interferon for Hepatitis C). It is possible that this patient's reaction was secondary to the Prolastin preparation due to PEG instead of the alpha-1-antitrypsin protein itself. Zemeira, which is another preparation for AAT augmentation, does not contain PEG and could be used if a true reaction to PEG was verified. Due to the temporal relationship of the rash with the Prolastin infusion and no other new medications, we believe that the Prolastin is the cause of the vasculitis. To our knowledge this is the first reported case of HV secondary to Prolastin administration. Physician and other healthcare professionals should be aware of the possible rare adverse reaction using Prolastin.

## Figures and Tables

**Table 1:** Pertinent laboratory tests.

Laboratory name	Laboratory Result	Reference Ranges
Hemoglobin	11.0	13.5–17.5 g/dL
Mean Corpuscle volume	90.2	81.6–98.3 FL
Leukocyte count	13.1	3.5–10.5×10^9^/L
Platelet count	222	150–450 ×10^9^/L
Creatinine	0.6	0.6–1.1 mg/dL
Blood Urea Nitrogen	26	6–21 mg/dL
Sedimentation rate	30	0–22 mm/Lh
C-Reactive Protein	23.9	<= 8.0 mg/L
Myeloperoxidase Ab	< 3.0	< 6.0 U/mL
Proteinase 3 antibody	< 3.0	< 3.0 U/mL
Cryoglobulin	Negative	
Cryofibrinogen	Negative	
Complement total	63	30–75 u/mL
Complement C1q	17	12–22 mg/dL
C3 Complement	150	75–175 mg/dL
C4 complement	20	14–40 mg/dL
Antinuclear Ab	0.9	<= 1.0 U
Anti DS DNA IgG	< 1.0	< 5.0 iu/mL
SS-A/RO Ab IgG	< 0.2	> or = 1.0 U
SS-B/LA IgG	0.4	> or = 1.0 U
Jo 1 Ab IgG	0.2	> or = 1.0 U
Scl 70 Ab IgG	< 0.2	> or = 1.0 U
Cyclic citrullated peptide	< 15.6	< 20 U
Anti glomerular basement membrane	< 1.0	<= 20.9 U
Alpha 1-anti-trypsin level	23	100–190 mg/dL
IgA	241	50–400 mg/dL
Hepatitis B antibody	Negative	
Hepatitis Be antigen	Negative	
Hepatitis B antigen	Negative	
Hepatitis C antibody	Negative	
Hepatitis C RNA detect/quant	Negative	

**Table 2:** Outpatient Medications.

Medication	Dose	Frequency
Albuterol/ipratropium		Q 4 hrs as needed for SOB
Alprazolam	0.5 mg by mouth	Daily at bedtime
Ascorbic Acid	1000 mg by mouth	Daily at bedtime
Bupropion	Unknown dose	At bedtime
Calcium/Vitamin D	500 mg/250 units by mouth	Twice a day with meals
Fluticasone/Salmeterol	500/50 discus inhalation	Twice a day
Furosemide	40 mg by mouth	Daily in am
Gabapentin	600 mg by mouth in am, 600 mg at noon, 1200 mg at bedtime	Three times a day
Glucosamine/Chondroitin	500 mg/400 mg by mouth	Daily
Guaifenesin SR	600 mg by mouth	Twice a day
Ipratropium MDI		Q 4–6 hrs as needed
Loratadine	10 mg (during allergy season)	Daily
Magnesium oxide	400 mg by mouth	Daily with breakfast
Montelukast	10 mg by mouth	At bedtime
Multivitamin	one tablet by mouth	Daily in am
Nasonex	1 spray to each nostril	At bedtime
Omega 3	1000 mg by mouth	Twice a day
Omeprazole	20 mg by mouth	Daily
Zoloft	100 mg by mouth	Daily
Sucralfate	1 gram by mouth	Four times a day before meals and at bedtime
Verapamil SR	240 mg by mouth	Daily in am

## Abbreviations

COPD: Chronic obstructive pulmonary disease
ANCA: Antineutrophil cytoplasmic antibody
TNF: Tumour necrosis factor
RF: Rheumatoid factor
AATD: Alpha-1-antitrypsin deficiency
AAT: Alpha-1-antitrypsin
HV: Hypersensitivity vasculitis
LV: Leukocytoclastic vasculitis
HIV: Human immunodeficiency virus
SLE: Systemic lupus erythematosus
RA: Rheumatoid arthritis
PeG: Polyethylene glycol
CSS: Churg-Strauss syndrome
SOB: Shortness of breathe.
